# How Women Discuss Heavy Menstrual Bleeding in Online Forums (“It’s Like Revenge of the Uterus”): Template Approach to Thematic Analysis

**DOI:** 10.2196/73532

**Published:** 2025-07-02

**Authors:** Emily Jade Young, Heather Mary Kirk

**Affiliations:** 1 Institute of Social Sciences Sheffield Hallam University Sheffield United Kingdom

**Keywords:** menstruation, heavy menstrual bleeding, medical sexism, medical dismissal, female health, reproductive health

## Abstract

**Background:**

Heavy menstrual bleeding (HMB) is a common condition that affects approximately 20% to 30% of women globally. However, despite significant physical, mental, and social impacts on the quality of life of women who experience HMB, they face barriers to both diagnosis and treatment. With current challenges to female reproductive autonomy growing on a global scale and with the stigma that surrounds menstruation, women with HMB may turn to online communities to access peer support and information. Online forums such as Reddit, which support the use of anonymous posting, may offer a space where those affected by HMB can share their experiences, seek support, and offer advice to others.

**Objective:**

This study aimed to explore how those experiencing HMB use Reddit to share experiential knowledge, provide support, and share experiences of HMB within an online community space.

**Methods:**

Data were collected from discussion threads on the *TwoXChromosomes* subreddit on Reddit. Publicly accessible posts were identified through a systematic search conducted on August 13 and 14, 2024, using keywords related to HMB. A template approach to thematic analysis was used to analyze the data. A priori codes were developed from existing literature on HMB and the research objective. The template was refined after further examination of the transcripts, with all transcripts being analyzed using the final template.

**Results:**

The search initially identified 434 discussion threads. Threads were screened for relevance and user engagement, resulting in a final 13 (2.99%) threads being analyzed for this research. These comprised 1505 individual comments from 1115 unique users. Four central themes were identified: validation and camaraderie, life impacts of HMB, practical support, and medical treatment and management. In the validation and camaraderie theme, users frequently shared personal experiences and validated the experiences of others, challenging the normalization of debilitating symptoms and creating a shared sense of solidarity. When discussing the life impacts of HMB, users emphasized how it disrupts daily functioning, including work, relationships, and mental well-being, and poses serious physical health risks. In the theme of practical support, Reddit users exchanged strategies for managing symptoms, including recommending specific menstrual products, home and workplace adaptations and adjustments, and self-advocacy. The final theme of medical treatment and management explored Reddit users’ frustration with health care experiences, particularly around the prioritization of fertility over quality of life. Hormonal contraception, intrauterine devices, and surgical interventions were discussed with varying degrees of satisfaction and concern. Overall, Reddit users reported a general dismissal of HMB within medical and social contexts.

**Conclusions:**

Reddit serves as an important platform for individuals with HMB to validate their experiences, share practical knowledge, and seek peer support in the face of medical dismissal. This research provides insight into the usefulness of online spaces for people discussing HMB.

## Introduction

### Background

Heavy menstrual bleeding (HMB), also referred to as menorrhagia, is a common reproductive condition affecting women and those born female worldwide. Approximately 27.2% of 4506 women across Switzerland, Germany, France, Spain, and the Netherlands self-reported HMB in a survey [1]. According to the National Institute for Health and Care Excellence, approximately 1 in 20 women in the United Kingdom aged 30 to 49 years visit their general practitioners (GPs) with concerns about HMB, with HMB accounting for 12% of all gynecology referrals [2]. In the adolescent population, up to 40% report experiencing HMB [3]. HMB is characterized by excessive or prolonged menstrual bleeding, defined by the National Health Service in the United Kingdom as the need to change tampons or sanitary pads every 1 to 2 hours or bleeding that persists for >7 days [4]. However, recent research has shown that this definition can result in an underestimation of the number of women who experience this condition, as modern menstrual products are more absorbent than previously assumed [5].

Several factors contribute to the onset of HMB. Hormonal imbalances, particularly fluctuations in estrogen and progesterone levels, can disrupt the normal menstrual cycle, leading to excessive bleeding [6]. Structural abnormalities of the uterus, such as fibroids [7], polyps [8], and adenomyosis [9], also increase the likelihood of HMB. Other contributing conditions may include endometriosis, pelvic inflammatory disease, and the use of certain medications [10]. HMB may also lead to iron deficiency anemia, resulting in symptoms such as fatigue, weakness, and shortness of breath [11]. In some cases, HMB occurs without a clearly defined underlying cause. Idiopathic menorrhagia, defined as HMB with no identifiable structural or hormonal abnormalities, can account for up to 50% of reported cases [12,13]. Treatment options for HMB are contingent upon the underlying cause, severity of symptoms, and patient preferences. Hormonal therapies, including oral contraceptives, progestins, or hormonal intrauterine devices (IUDs), are frequently offered to those with HMB to reduce overall bleeding [14]. Nonsteroidal anti-inflammatory drugs may also be suggested to alleviate pain and reduce menstrual blood flow [15]. For individuals with structural abnormalities such as fibroids or polyps, surgical procedures such as endometrial ablation or myomectomy may be considered [16]. In cases where these measures are ineffective or in instances of severe HMB, a hysterectomy may be suggested [17].

Despite the availability of treatment options, many women remain undiagnosed [18]. This may be due to patients’ reluctance to discuss menstrual health issues because of the stigma associated with periods and bleeding [19] or a lack of awareness or training among health care providers regarding HMB [20]. Furthermore, HMB is often misconstrued by women as “normal,” leading to underestimation and underreporting during discussions with medical professionals [21]. Cultural taboos, societal norms, and misinformation all contribute to the stigma surrounding menstruation [22]. Such stigma may cause feelings of shame and isolation among those experiencing HMB [23]. Misconceptions about menstruation may also lead to dismissive attitudes from health care providers, creating barriers for necessary treatment and support [24,25]. HMB may also be misdiagnosed, with women reporting that their GPs often fail to recognize HMB based on self-reported symptoms [26]. A qualitative study of women diagnosed with HMB for a decade revealed mixed experiences; while some reported positive interactions with health care professionals, others felt dismissed by their GPs [27]. As a result, this can exacerbate the impact that HMB can have on an individual’s well-being [28].

HMB has been shown to significantly impact an individual’s physical health, emotional well-being, and overall quality of life [29]. A study of 1547 women in Sweden found that 32% reported experiencing HMB, which was significantly correlated with a decreased quality of life [30]. In another multinational study of 6179 women, self-reported HMB was associated with negative impacts on social life, relationships, and occupational functioning [31]. In addition, women with HMB frequently report feelings of embarrassment, anxiety, and isolation due to HMB [32]. While existing research has focused on understanding the experiences and pathology of HMB, there has been limited exploration of the role of social support and knowledge sharing among those affected by this condition.

Because of the stigma and reluctance to discuss menstrual bleeding, many women may turn to the internet for information and support, as it provides a convenient, asynchronous, and anonymous way of discussing personal and stigmatizing conditions [33]. Women may use social support mechanisms, such as online groups, as a means of understanding and making sense of their condition and symptoms. Social media sites can provide users with the opportunity to seek out advice and discuss treatments for stigmatized conditions in an anonymous way and can help create a supportive community of individuals with shared experiences [34]. However, this has yet to be explored within the context of HMB. Gathering experiential information that is created by women is essential to pinpoint priority areas where health care professionals could provide support and guidance [35] and to understand what support these individuals need from health care systems [36]. Social media and online forums offer a promising source of patient-centered insights. In addition, because HMB is often viewed as a stigmatizing condition, individuals may be reluctant to participate in research studies due to embarrassment [37]. Therefore, more naturalistic data may provide insights that typical data collection methods might omit [38]. Infodemic methodology offers a framework for studying and analyzing health-related information disseminated through online platforms [39]. These are naturalistic data, and the use of anonymous accounts allows users to post comments and discuss HMB more freely.

### This Study

This study aimed to analyze online discussions among women on Reddit to explore how women share knowledge and experience of HMB, as well as to understand the support users may offer each other. Similar methodologies have been applied to other stigmatized health phenomena, including breastfeeding [40], erectile dysfunction [41], miscarriage [42], and endometriosis [43]; therefore, it provides a useful approach to understanding how women discuss HMB and the sort of support they seek from other individuals with the condition.

## Methods

### Study Design

The study uses an infodemic approach, which involves using online data to investigate the dissemination and exchange of health-related information [39]. Reddit was chosen for this study due to its high engagement and anonymous posting format. While menstrual disorders are also discussed on other online platforms (eg, Facebook, X, and forums such as Mumsnet), Reddit’s structure allows for large-scale, anonymous discussions, which makes it suitable for exploring sensitive health experiences such as HMB. The *TwoXChromosomes* subreddit was selected because it is a popular, highly active forum that attracts a female-focused community where users frequently discuss reproductive health topics, including menstruation. As some Reddit users self-identified as transmen and nonbinary, this paper will refer to participants as “reddit users” to adopt an inclusive approach. A systematic search was conducted using the terms “heavy menstrual bleeding,” “heavy period,” and “menorrhagia.” These terms were chosen as they reflect both clinical terminology and everyday language commonly used in academic literature, health care settings, and social media discussions. EJY completed the search on Reddit on August 13 and 14, 2024. Threads were initially screened by reading titles and opening posts (OPs) to determine whether HMB was the central focus. The second stage of screening involved reading the full threads (OP and comments) to ensure the relevance of the data. To help mitigate the risk of inauthentic content, only threads with >5 unique contributors were included. Posts that were >10 years old were excluded to ensure the data reflected modern accounts of HMB. All final threads were reviewed by HMK to ensure they met the inclusion criteria. Any threads where eligibility was unclear were discussed between EJY and HMK until an agreement was reached.

### Ethical Considerations

Ethics approval was granted by Sheffield Hallam University (ER65866143), and this research adheres to the British Psychological Society’s guidelines for internet-mediated research. Publicly available discussions were analyzed, ensuring compliance with the British Psychological Society’s internet-mediated research guidance [44]. Consistent with recommendations from Eysenbach and Till [45], informed consent was not obtained due to it being a freely available public forum and accessed without subscription, and the anonymous nature of forum users. However, usernames were anonymized, and any identifying information was redacted for confidentiality purposes.

### Data Analysis

A critical realist and feminist epistemological stance underpinned this research. Therefore, this research recognizes the reality of women’s experiences with HMB while acknowledging that the discourse around HMB is socially constructed, particularly with the gendered dynamics of female reproductive health.

Discussions were anonymized, with usernames being replaced with the transcript number (T1-T13) and the Reddit user number assigned based on the first comment in that particular thread (eg, RU1). All data were uploaded into NVivo (version 12; Lumivero) for analysis. A template analysis was conducted, following the methodological framework outlined by King [46], as it is suited for data generated for different purposes [40]. The process began with familiarization, and all discussion threads were thoroughly read and reread to gain a comprehensive understanding of the discussions. A priori codes, derived from the research questions and previous research on HMB, are detailed in Multimedia Appendix 1. The first coder (EJY) coded the data using this initial template, with adjustments made based on developing codes. A secondary coder (HMK) independently coded 2 discussions. Amendments to the template were made following discussions between EJY and HMK. The final template was applied to all discussions by EJY and subsequently double-coded by HMK to ensure triangulation and agreement. Codes were then discussed between EJY and HMK and grouped into broader categories, which were developed into themes.

### Reflexive Statement

EJY is a 35-year-old woman with endometrial hyperplasia and experienced medical dismissal of HMB for 20 years before receiving treatment. EJY believes HMB is poorly understood and undertreated by the National Health Service in the United Kingdom. A reflexive journal will be kept during data collection and analysis to ensure greater objectivity.

HMK is a 53-year-old perimenopausal woman who has experienced normal menstrual bleeding throughout her fertile years. HMK kept a reflexive account throughout the analytic process, and issues around HMB were discussed with EJY to ensure transparency in analysis.

## Results

### Overview

**Figure 1:**
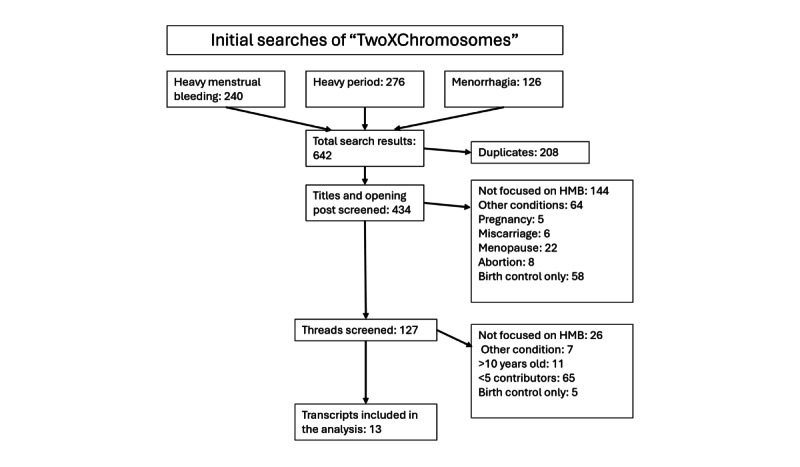
Search strategy. HBM: heavy menstrual bleeding; OP: opening post.

A systematic search was conducted using 3 key terms: “heavy menstrual bleeding” (240 discussions), “heavy period” (276 discussions), and “menorrhagia” (126 discussions). Duplicate discussions were identified and removed (208/642, 32.4%), leaving 434 (67.6%) discussions. These were initially assessed by reading the title and OP and screening out discussions that did not focus on HMB, resulting in 127 (19.78%) remaining discussions. Further exclusion criteria involved removing posts that lacked sufficient focus on the topic (n=38, 5.92%), had <5 unique contributors (n=65, 10.12%), or were >10 years old (n=11, 1.71%; Figure 1). Finally, 13 (2.02%) discussions remained for analysis, comprising 1505 comments from 1133 unique contributors (Table 1).

**Table 1:** Discussion threads used in the analysis.

Thread	Time since post	Unique contributors (n=1133), n (%)	Comments (n=1505), n (%)
Thread 1	6 months	11 (0.97)	19 (1.26)
Thread 2	6 months	7 (0.62)	32 (2.13)
Thread 3	4 years	155 (13.68)	217 (14.42)
Thread 4	5 months	26 (2.29)	47 (3.12)
Thread 5	3 years	214 (18.89)	262 (17.41)
Thread 6	8 months	139 (12.27)	187 (12.43)
Thread 7	2 months	40 (3.53)	44 (2.92)
Thread 8	1 month	18 (1.59)	35 (2.33)
Thread 9	6 years	18 (1.59)	46 (3.06)
Thread 10	2 months	27 (2.38)	31 (2.06)
Thread 11	3 years	13 (1.15)	33 (2.19)
Thread 12	2 years	454 (40.07)	522 (34.68)
Thread 13	1 years	11 (0.97)	30 (1.99)

Following a template approach to thematic analysis, 4 themes were identified: validation and camaraderie, life impacts of HMB, practical support, and medical treatment and management. Throughout all themes, Reddit users discussed diagnosis, treatment, and the experience of having HMB.

### Validation and Camaraderie

This theme explores a shared camaraderie among the Reddit posters. Reddit users offered validation as support for what others were going through. They tended to share their own experiences to create a shared experience, camaraderie, and shared understanding of HMB. This was sometimes in the essence of “you are not alone,” but most often it was to communicate that debilitating symptoms were not a normal part of womanhood that should be accepted:

I had a similar experience. Took about 3 years of constant bleeding, a bad infection and multiple blood transfusions to be taken seriously.

T12RU58

This validation was used to help other Reddit users understand what normal, healthy menstruation should be like:

It’s so sad how many women live with symptoms like this not realizing this isn’t normal and shouldn’t be ignored.

T10RU6

Users highlighted how the availability of social media was helping to educate women on what was, and was not, abnormal, as pointed out in the following quote:

I hope social media has opened the eyes of women everywhere and allowed us to be open and honest in search of understanding and acceptance, but that it’s also inspired its users to be the same for their family and friend groups so the new generations don’t live with the shame and misinformation our generations were given.

T10RU5

This was also linked with how women define HMB. While the medical community ascribes quantitative measurements, the definition of HMB among Reddit users was rooted in experience, imagery, and affect:

I always knew my periods were awful because my bathroom looked like a Dexter kill room.

T3RU78

Reddit users often used humor throughout these discussions. This seemed to serve several functions: to bring some form of lightness to otherwise quite difficult conversations, as a coping mechanism to enhance well-being in the face of distressing experiences, and as a means of creating affiliation with others. Humor was used by multiple users, most often in the form of euphemisms and analogies:

Yeah, if they ever luminol my bathroom I’m going to jail while they excavate my yard for the obviously dozens of murder victims.

T3RU24

Validation did not always just come from other Reddit users, it was also something that some users identified in real life. This could be from a romantic partner taking their symptoms seriously, a friend supporting them, or a supportive workplace:

In college I had a professor that let us stay home and not attend class if we had bad periods.

T9RU1

This validation and camaraderie would then usually lead to suggestions for further testing. Reddit users frequently drew on their own experiences to recommend specific diagnoses to consider and tests that a user could request from their physician. This was most often related to endometriosis, hyperplasia, and fibroids. However, sometimes, less obvious potential causes for HMB would be suggested:

This is gonna sound random but have you had your thyroid checked? I was bleeding this much and it turned out I have hypothyroidism.

T5RU106

These types of discussions were aimed at educating and empowering other users to continue to pursue answers for their HMB and identify the root cause of the HMB. Overall, validation was used as a way to support other users by affirming that their experiences were not typical and should not be dismissed as normal. Camaraderie was used to create a sense of shared belonging for a topic that is usually isolating.

### Life Impacts of HMB

Reddit users discussed the impacts of HMB on their everyday lives. These included experiences of living with HMB symptoms for years—sometimes even decades. Many users who had now received adequate treatment for HMB reflected on the years they had endured its symptoms::

After nearly 15 years of hell, they removed my uterus.

T12RU23

Struggling for years with HMB led to some reporting serious effects. One user, who identified herself as working in a pathology laboratory, recalled seeing the notes on a specimen of a woman whose endometriosis had led to her “dropping out” of college. One user described HMB as being forced to live for >10 years with torture. Another user reported that treatment came too late:

It took me over 20+ years to get diagnosed. By then it was too late, and now I have massive complications from how far my endo went. I’ll be in pain management the rest of my life. Thank you to all the doctors who ignored me in my teens, 20s, 30s...

T5RU159

HMB has such an impact on users’ lives that they discussed suicidal ideation, and users discussed wanting to end their lives rather than live with HMB any longer:

I feel at a loss...I cannot continue to live like this.

T2RU1

My uterus is making me deeply suicidal...There are women who are on multiple antidepressants and recovering from suicide attempts due to their condition.

T5RU1

Along with thoughts of suicide, users seemed to be genuinely terrified of what was happening to their bodies. Some users talked about the lack of control over their own bodies, along with fears that the medical community was misdiagnosing them or overlooking potentially life-threatening conditions. Users discussed living in a state of anxiety due to HMB:

I’m terrified. I’m terrified, i’s all pretty heavy bleeding, and cramping, and just will not stop...I feel like utter garbage.

T7RU1

In addition to mental health impacts, users also described physical health consequences. Many users reported developing anemia due to excessive blood loss—some to the extent of requiring blood transfusions:

Turns out I was bleeding to death through my period. YES, my period was killing me. I had to have 3 blood transfusions.

T9RU1

Alongside anemia, many users discussed symptoms such as dizziness, including several accounts of users “passing out” at school, places of work, and home. Further physical effects included fatigue, fainting, cramps, migraines, and more:

Cramps, back pain, pain down my thighs, hot flashes, vomiting, extreme fatigue, menstrual migraine...even with ibuprofen.

T10RU1

The effects of HMB impacted users’ social interactions and day-to-day activities. One user discussed having to cancel a skiing trip due to HMB and others discussed social events they had to cancel. Some users found just attending work difficult due to HMB:

I remember having to call off work because of my period. People thought I was making it up but no, I literally couldn’t leave the bathroom because of the constant bleeding. It was bad and I don’t wish it on anyone.

T12RU74

These impacts also affected personal relationships, with users discussing the impact of HMB, particularly in relation to their sexual relationships:

A female gynecologist and she LAUGHED at me when I said I had never had sex. Of course I haven’t! There’s a river of blood constantly erupting out of my uterus! How am I supposed to explain that to a partner!

T5RU124

One of the most commonly discussed effects on life was “leaks,” where menstrual products failed, resulting in menstrual blood being visible on clothes, furniture, and other surroundings. Users discussed the emotional impacts of this, particularly embarrassment. One user reflected on an experience while still in school:

I was in my history class in middle school and asked to go to the bathroom and my (male) teacher wouldn’t let me go. I asked three times and finally started crying and he let me go begrudgingly but it was too late, I had bled all through my pants and onto the chair. One of the boys in class saw and loudly yelled “Ew! There’s blood on the chair!”

T11RU4

There were several examples given of situations in school and work where users had “leaked,” causing them embarrassment. Some users discussed changing their routine to adapt to HMB, with some working from home so they could sit on a towel during heavy bleeding, while others just stayed home during the heaviest days of their period:

I would stay home on the first 2 days of my period, not because of pain but because my jeans would have blood stains after only 2 hours.

T9RU3

The combined mental and physical effects of HMB left some users feeling hopeless:

I’m not here for HELP. Cause I already realized there is no help. I’ve tried everything.

T13RU1

Overall, HMB impacts the physical, social, and mental health of individuals. This can cause suicidal feelings, feelings of anxiety, and hopelessness. The stigma surrounding menstrual blood also contributes to feelings of embarrassment, causing those with HMB to miss social events and isolate themselves.

### Practical Support

Practical support was offered through users providing advice to women on managing the experience of HMB. This included discussions about menstrual products and recommendations for the best way of containing HMB. This included comparisons and discussions around tampons, maternity pads, period pants, and menstrual cups, along with the efficacy of particular brands in managing HMB:

Yes!! I use thinx with a heavy pad and I finally don’t have to worry about bleeding on the bed while i sleep!!

T3RU31

However, users also advised other users about poor quality menstrual products and usability, particularly around menstrual cups, which were seen as messy and impractical for work. Some users discussed the inadequacy of menstrual products, even those designed specifically for HMB:

Before bc I would double up on super plus tampons and maxi pads during the day and flood out of them in a couple of hours. Not “a bit of leakage” I’m talking ruining jeans, chairs, the works.

T12RU339

Along with advice on menstrual products, users offered further advice in terms of protection from HMB and potential leaks. These involved the use of incontinence pants in addition to menstrual products, as well as offering practical advice on clothing:

I’m sorry for the underwear you thought you could get clean. Think in dark colors now, it’s safer.

T3RU117

Most often, users discussed the “double up” method, combining menstrual products to maximize coverage and minimize the chances of blood seeping through:

I used to double up on tampons and pads, because I would bleed through a tampon in an hour, especially the first day.

T1RU5

However, users also offered wider practical advice to manage potential leaks. Many users agreed on ensuring wipeable flooring in the bathroom as well as ensuring there were no bathmats near the toilet itself. Some discussed using towels to protect furniture, while others suggested the use of puppy training pads attached to sofas and beds to protect furniture:

Have you tried disposable “pee”’ pads for your mattress? They do get mussed up when you move around, but maybe you could pin them in place or something. It might mean less sheet washing.

T1RU2

Further advice was offered around nontraditional remedies. This included acupuncture, naturopath appointments, dietary changes, as well as different vitamin combinations. Some users discussed vegan diets as improving their symptoms, and others spoke avidly about Chinese herbal medicine. Various combinations of vitamins and supplements were offered as useful:

Homeopathic Sabina drops 30 CH strength, take 5 drops on the tongue 3-5 times a day of your period until you’re happy with the flow amount.

T11RU13

However, several Reddit users expressed skepticism toward nontraditional medicine, voicing concerns about the exploitation of people in vulnerable situations:

Gynecology is RIPE for snake oil creeps to miseducate and take advantage of people that don’t fully understand the physiology desperate to find a solution to the issue their having that may also not trust conventional medicine.

T2RU2

Users also shared resources and book recommendations. These resources included books such as *The Period Repair Manual*, as well as wellness blogs, news articles, and academic literature:

Our Bodies, Ourselves is an excellent book on women’s health and sexuality.

T12RU299

Users also offered practical advice on self-advocacy. As discussed in a later theme, users often highlighted poor attitudes by the medical profession and felt “fobbed off” when seeking help. Other users highlighted how those attending medical appointments could advocate for themselves. This sometimes involved creating accountability by refusing to be discharged unless the medical professional provided a diagnosis or listed the symptoms and signed their name to agree there was no health risk:

Tell them you want it in writing. Doctor is refusing you a medication? Please write down your reasoning, sign, & date, Doctor! :)Doctor is refusing you a medical procedure? Please write down your reasoning, sign, & date, Doctor! :)

T6RU23

Others advocated for sharing the names of physicians who failed to provide adequate care:

Start a fucking witch hunt against these assholes, report them to any type of medical board, just constantly report each time we get told no because apparently our health doesn’t matter.

T5RU124

One user who talked about only receiving help once she physically bled through her clothes in the emergency department of the local hospital, advocated for more extreme measures. This comment demonstrates the clear frustration with the need to self-advocate:

I’d be saving all my used period products, bled through sheets, clothes and printed out photos of the puddles(?!?!?) and dumping them on the front desk of the ER like “IS THIS ENOUGH BLOOD FOR YOU?!”

T8RU2

Overall, Reddit users shared practical sources of support, including clothing tips, menstrual products, alternative treatments, and self-advocacy strategies, highlighting the issues they found most impactful in their daily lives.

### Medical Treatment and Management (Hormonal Birth Control, IUDs, and Surgical Options)

#### Overview

Reddit users shared their experiences with the medical treatment and management of HMB, focusing on the physical and psychological effects of various treatments, including hormonal birth control (HBC) pills, IUDs, and surgical options. Discussions also highlighted how interactions with health care professionals shaped users’ well-being, often leading to frustration and a loss of trust in treatments. Users shared their experiences to support others, understand discrepancies in their own experiences, and offer advice on what had been effective for them.

#### HBC Treatment

Many users discussed their experiences with HBC as a treatment for HMB. They talked about how HBC, as a method of treatment, affects their physical and psychological symptoms that they associate with HMB. For some users, HBC seemed to provide relief by reducing their bleeding:

I went for the mini pill (progesterone only) and now no longer have periods at all and it is glorious.

T4RU5

Others talked about the relief of having a marked improvement in their mental health because of HBC:

I got on bc this year and it’s been wonderful. It’s predictable, less moody like I’m not trying to get my ass kicked in a bar fight, less depressed and generally it goes by a lot smoother.

T3RU13

However, HBC did not seem to have a universal beneficial effect. Many users reported that HBC seemed to exacerbate symptoms and make them feel physically and mentally worse than before treatment. For example, users described experiencing worse physical side effects while on HBC compared to being off it, including continuous bleeding and increased pain:

I was on a new pill at an absurd dose that was making me a basket case, increasing the pain, and I’d already been bleeding for three months straight.

T5RU26

Others stated how the hormonal changes with HBC negatively impacted their psychological well-being:

The birth control wrecked my body and hormones, and also caused me to lose my mind (hence the counselor).

T5RU195

Some users felt that they had to choose between HMB and the side effects of HBC, both of which severely impacted their quality of life. This decision involved weighing up the pros and cons of taking HBC against the symptoms that they were already experiencing. For some, this decision meant that they would rather put up with HMB than take HBC:

*hormonal BC can wreak havoc on a body, and I have enough going on without that anyway. More to thepoint, I don’t want to take it.* [T5RU7]

For other Reddit users, it was the perceived weight gain that they found the most challenging aspect of using HBC and influenced the decision to use HBC or to continue taking HBC as a way to control HMB.

*I want off hormonal BC because I literally can’t loose weight while on it. I finally managed to startloosing weight when I was off it for 3 months.* [T5RU28]

Some users reported that they had persevered with HBC and tried several different types of HBC before they were satisfied with the outcome effects on their HMB, implicitly suggesting that it is worth trying different options:

I will say when I first started on the pill years ago, it took trying 3-4 different ones to find one that I was happy with and since then the one I was on stopped working well and I had to switch to something else.

T4RU13

Many users expressed concern that HBC was often the only treatment offered for managing HMB, with the medical profession perceived as unwilling to explore alternatives. They felt their experiences of side effects were dismissed, leading to feelings of being unheard or ignored by physicians. Some were repeatedly told to “wait and see,” or their symptoms were minimized as “normal” or “common.” One user stated the following:

I bled on the pill for 3 months straight and the gynecologist just said those are “common symptoms” and to “give it time.” I eventually just went off because I felt so terrible on it.

T6RU63

This resulted in patients either stopping treatment themselves or finding their own treatment options. This demonstrated that the users felt that they had to deal with things on their own because of their symptoms being dismissed. Another user stated the following:

I put myself on birth control to ease my periods, because the doctors kept putting me on ones that messed me up more so I had to do my own research and sort it myself.

T5RU86

This suggests that some users, feeling unheard by medical professionals, see turning away from medical advice as a viable option and instead begin seeking out their own treatment solutions. Others seemed to wish that they could find someone who would listen and take their experiences with HBC seriously:

I just want to find a doctor who will listen and perform the right tests and not just slap BC as the solution in my face. BC landed me in the ER and now I’m scared of everything hormonal 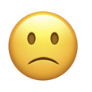.

T10RU1

#### IUD Treatment

When discussing treatment options, many users described how the IUD affected them physically and the psychological impact of having one fitted. Many of the users described their reactions to being prescribed the IUD and their beliefs and feelings toward what can be considered an invasive procedure. Many Reddit users were wary of having an IUD fitted and had fears about how it might affect them. This seemed to lead to a lack of trust in the treatment effectiveness and concerns about how it might affect their psychological well-being:

I feel uncomfortable on the IUD for a few reasons...Not knowing how I will react to it once it is inside me. Some people tolerate it alright, some people get very depressed, break out, gain weight, lose they’re sex drive—overall just feel like a different person...the idea of not getting any relief at all for that procedure seems inhumane to me. And that makes it hard for me to trust this process entirely.

T2RU1

Many users discussed their symptoms being worse following the IUD insertion, as well as discussing complications from IUDs going “missing.” For some users, while their bleeding improved, they experienced other symptoms, such as cramps described as “debilitating,” headaches, and hormonal surges. For some users, the bleeding worsened after getting an IUD:

Got mine removed because I was in misery for two years. Periods were bloody, painful, and longer. I also got cramps pretty much constantly.

T3RU54

However, for some Reddit users, IUDs were an answer to reducing HMB. Some users described the IUD as a “miracle,” with a few noting their intention to have it replaced as soon as possible after pregnancy:

Now I have an IUD and basically no periods at all.

T9RU10

Some users talked about the relief they felt at having their bleeding stop entirely because of their IUD:

I got the hormonal IUD and voila; after really heavy bleeding right after IUD insertion I went down to no periods at all. I was in heaven.

T12RU324

However, pain was a commonly reported symptom by the users, even when the IUD was reported to help with the bleeding. Some users engaged with vivid imagery to help them describe and convey how the pain felt for them:

I have an IUD so I don’t have periods anymore thankfully but several times a month my uterus decides to snuggle a steak knife in and try to escape.

TR5U93

Some Reddit users described the pain as a side effect of having the device rather than their HMB. In some cases, the pain was so severe that they chose to have the device removed, ultimately discontinuing this form of treatment:

The only one that worked was the IUD but that thing felt like I had a tiny pissed off demon in my uterus clawing to get out. After about 2 years of that I gave in and had it removed.

T9RU14

Therefore, there were mixed experiences and expectations regarding the use of an IUD to treat HMB.

#### Surgical Options

When discussing surgery, users typically outlined specific medical procedures that had successfully helped their HMB or that they believed would help their HMB. They typically related their surgical beliefs and experiences to their particular diagnoses. Those who had received surgical intervention often found relief, with the main surgical interventions being ablation (cauterization of the endometrial lining) or hysterectomy (removal of the uterus). Both surgical options were reported to have been successful, although users acknowledged that ablation surgery was a temporary solution:

I had a uterine ablation in November, and it’s been the best thing ever. Yes, I understand it may come back, and I’ll ultimately need a hysterectomy, but so far, so good.

T12RU61

Hysterectomies were reported as lifesaving, with users reporting relief following the removal of the uterus. Users did discuss the side effects of a premature menopause; however, these fears were offset by surgeons leaving the ovaries in situ. Users talked about hysterectomies as being the best decision they could have made, with some referring to it as the best day of their lives:

I tell everyone who asks, that as much as I love my kids, the day I got my hysterectomy was the happiest day of my life.

T12RU73

However, most users expressed their frustration at the limitations in accessing these surgeries, with users feeling like they had to fight for medical intervention. One user discussed the life-threatening implications of these limitations, referencing her aunt’s experiences with the surgeon’s decision to wait to see if natural menopause would cure the HMB:

My aunt was like that and almost died—she wound up needing a hysterectomy, but she had to wait 6 weeks of solid bleeding and blood transfusions because she was so close to menopause and they were seeing if it would stop on it’s own.

T12RU28

Many users discussed medical professionals prioritizing other things such as fertility over the treatment and well-being of the patients. This caused frustration and anger, with users left feeling their life was less important than their potential ability to carry a child for a man, even if they were adamant they never wanted children:

I have never wanted children. Ever. I am an antinatalist for fucks sake but my wants and needs don’t matter. My life doesn’t matter, because maybe just maybe, a man can impregnate me and I can be a cute little incubator.

T5RU1

Even users who already had children outlined that they had been denied surgical options, with surgeons reported to be concerned that a user may “change their mind” and want further children in the future. Some users had husbands who advocated for them, with some surgeons agreeing to surgery once assured by the husband that there were no further desires for more children. However, this did not always work; one user discussed being denied surgery despite having children and her husband having a vasectomy, with reference to a hypothetical scenario that may never happen:

When I approached my doctor about a hysterectomy and I told him my husband had a vasectomy, he said “well what if you get divorced and want another child with a different man?” The desires of a non-existent man were made a higher priority than my own rights.

T12RU75

Overall, users discussed their experiences with the medical treatment of HMB to share information about potential side effects, create a shared understanding of the treatments, and express their frustration and anger at feeling unheard or dismissed by medical professionals. While hormonal contraception worked for some users, it was often the only method relied upon to manage HMB. This meant that for those who found hormonal contraception to not work or to worsen symptoms, very few options remained. For IUDs, it seemed that while the users experienced a reduction in bleeding, some were fearful of having the device inserted, and others reported pain continuing because of the IUD. With surgical options being reported as life-changing, many users believed that they were denied these treatments because preserving fertility was prioritized over their quality of life.

## Discussion

### Principal Findings

This study explored Reddit users’ experiences and perspectives on HMB using a template approach to thematic analysis. Reddit enabled the identification of in-depth discussions about HMB—insights that may not have been accessible through other research methods [33]. This provides a novel insight into the sharing of knowledge and experience of HMB, as well as the support users may offer or seek. The aim was to explore how women share knowledge and experience of HMB, as well as to understand the support users may offer each other. Four key themes were identified in this study: validation and camaraderie, life impacts of HMB, practical support, and medical treatment and management. Overall, this study suggests that online spaces may facilitate anonymous discussion of HMB, which may lead to empowerment through shared knowledge and collective support to those experiencing HMB. Findings suggest that the medical, emotional, and practical needs of those with HMB may not be met in health care systems, but rather through online peer support.

### Comparison to Prior Work

Validation and camaraderie reflected how the Reddit users supported each other’s experiences. They tended to present their own experiences to create a shared experience, camaraderie, and a shared understanding of HMB. This was often intended to communicate to others that the debilitating symptoms of HMB should not be accepted as a normal part of womanhood. This seemed to create an understanding of what normal, healthy menstruation should be like so that other users can compare their experiences to determine whether they need to seek treatment and to empower other users to continue to pursue answers for their HMB. Previous research has shown that due to the stigmatization of menstrual bleeding, women tend to be unaware that their experiences of menstruation may be abnormal [47]; therefore, turning to Reddit forums can be one way of educating themselves and others about the seriousness of symptoms. Sharing experiences in an anonymous manner on a Reddit forum can help reduce stigma and foster a sense of camaraderie and community [48]. When sharing these experiences, users frequently used humor to lighten difficult conversations and create a sense of camaraderie and belonging. It can be argued that humor is also used as a way of dealing with the more serious and scary aspects of HMB, thus helping the person posting the humorous comment to deal with their situation and to normalize the way they feel about their everyday experiences [49]. Previous research has shown that humor can relieve stress and help improve a person’s overall quality of life [50]. In this research, the use of humor facilitated discussions that centered on medical dismissal and reported severe impacts of HMB on quality of life. Research has previously identified humor as an effective emotional coping strategy for those dealing with stigmatized illnesses, such as HIV [51] and testicular cancer [52]. This also ties in with some of the suggested humor and sarcasm relating to health care provider interactions, as humor can help individuals to discuss stigmatized health issues with their health care providers [53]. Therefore, humor may serve a dual role—as both a coping mechanism and a form of resistance to stigma.

Reddit users discussed the impacts of HMB on their everyday lives. These included experiences of spending many years living with the symptoms of HMB. The users reported that HMB impacted their daily lives—at times to a life-threatening degree—either due to excessive blood loss or suicidal thoughts resulting from the ongoing burden of symptoms. These impacts included restrictions on social activities, work, and sexual relationships and negative effects on emotional well-being through isolation from embarrassment, anxiety, and stigmatization. Users seemed to be very frightened of what was happening to them. Some users talked about the lack of control over their own bodies and feared that the medical community was not taking them seriously or treating them in the most appropriate way. Previous studies have shown that HMB can cause serious physical disruption and result in several physical health complaints, such as severe anemia, fatigue, weakness, and breathlessness [54]. This level of physical debilitation can lead to disruption in daily functioning for these users. This level of disruption can lead to detrimental impacts on interpersonal relationships and social well-being [55]. Indeed, the users reported that because of the physical effects of HMB, their social functioning was impaired, with an impact on the ability to work, take part in fun leisure activities, and interpersonal sexual relationships. Previous studies have shown that negative experiences with menstruation can lead to an impairment of social well-being [56,57]. This is compounded by the emotional impact that HMB can have through the hormonal changes experienced during menstrual cycles, with some users reporting this to be one of the most disruptive and distressing aspects of the HMB experience, which is in line with previous findings [27,58]. However, previous research identified that within a medical context, health care professionals prioritize blood loss above other symptoms, rooted in the biomedical model of health [55]. These findings suggest that the lived experiences and impacts of HMB transcend this biomedical framework, suggesting that the full extent of individuals’ challenges may not be adequately reflected in clinical or diagnostic criteria. Therefore, a biopsychosocial approach to HMB may serve the affected population best.

Practical support was evidenced by users providing advice to women on managing the experience of HMB. This included discussions around menstrual products, recommendations for the best way of containing HMB, and using clothing in ways to reduce the chance of leakage. Previous research has shown that leakage of blood during menses is a cause of anxiety, embarrassment, shame, and guilt for women [59] and that this is exacerbated when the blood flow is heavy [60]. Research has also shown that online spaces can offer experiential knowledge without the risk of negative interactions that may occur in real-life settings around stigmatized health behaviors, such as breastfeeding, miscarriage, and sexual health [42,61,62]. Users also provided advice on managing the physical effects of the symptoms, such as alternative therapies and remedies, to help alleviate the biological and psychological effects, further sharing experiential knowledge. Users also shared practical advice for trying to achieve a diagnosis for the causes of HMB by suggesting specific tests or diagnosis that other users can discuss with their health care providers. Previous studies have shown that this is a common practice for individuals with stigmatizing conditions with vague symptomology that can be attributed to many different conditions [37]. This is important because patients who attend health care appointments well prepared with clear outlines of symptoms and suggestions can have more satisfying interactions with health care providers [63], resulting in mutual trust and shared decision-making [64]. However, this is not always the case, as some research has shown that individuals who do not receive the answers that they were anticipating from their health care providers can end up more dissatisfied with the patient-practitioner relationship and, as a result, are less adherent to medical advice [64,65].

Some users focused their practical advice on guiding other users on techniques to persuade medical professionals to take them seriously. This links with previous research suggesting social media may act as a tool for reproductive health activism, with research showing that platforms such as Instagram (Meta Platforms, Inc) are used to educate women about endometriosis, challenge the systemic sexism, and dispel myths [66]. While persuasive techniques often focused on overcoming barriers to diagnosis, there was a general call to action within discussions to all users to loudly and publicly demand better care for female reproductive disorders. This is similar to the online calls to action following the overturning of *Roe v Wade* in the United States [67,68]. Online discussion forums can offer valuable support and empowerment; however, they cannot substitute for professional care and frustration occurs when health care systems fail to keep pace with community experiential knowledge.

Reddit users also discussed medical treatment and management of HMB. This was often discussed as a frustrating and difficult topic, with conflicting narratives about the use of HBC, IUDs, and surgical options. Users often mentioned that HBCs were offered as an initial way of treating HMB. While for some users, HBC did reduce their symptoms, this was not a universal experience, with some users experiencing worsening or even new symptoms. This is similar to existing literature; while some studies have found that the combined contraceptive pill reduces HMB [69], others suggests it has no significant effect [70]. In addition to the effectiveness of HBC, its acceptability among users is also a key consideration. Users reported that HBC was presented as the default, sometimes only, treatment option with little regard for individual symptoms or patient concerns. These findings are supported by existing research highlighting the various reasons why many women may find HBC unacceptable, including side effects, lack of symptom relief, concerns about long-term use, and a desire for nonhormonal options [71]. There were similar experiences related to the use of IUDs, with some users reporting relief from HMB, while others reported worsening symptoms. As with HMB, those who had an IUD and were unhappy reported difficulties in getting medical professionals to listen to them, echoing previous research [72].

Reddit users were more positive about surgical interventions, which often yielded unanimous relief from HMB; however, access to surgical options was limited, with health care professionals prioritizing fertility over surgical treatment. Medical sexism is not a new topic; however, it is understudied. For women, the choice to become infertile through surgical procedures is still a taboo position that directly conflicts with the dominant social narrative of motherhood being central to the female experience [73]. In this study, even users who already had children, or for whom circumstance, sexuality, and choice meant they did not want future children, the opportunity for surgical intervention was still denied. There is a body of literature that explores sexism in female reproductive health care around HMB [74,75]; however, more research is needed. Overall, this theme suggests that there is an urgent need to restructure care around HMB to a patient-centered approach that prioritizes the individual’s autonomy and quality of life, recognizing diverse treatment goals and the ability of individuals to provide informed consent.

### Implications for Practice

The findings of this study have key implications for health care professionals who encounter women with HMB. The first implication is the limited recognition of HMB symptoms beyond blood loss, overlooking the broader range of physical, emotional, and social impacts. Health care professionals should take a biopsychosocial approach to the management of HMB. This is supported by previous research that highlights how a biopsychosocial approach to premenstrual disorder and menopause can improve patient outcomes and patient-physician interaction [76,77]. In addition to adopting a biopsychosocial approach, health care professionals should seek to elevate the medical autonomy of women reporting HMB, aligning with a more patient-centered approach. This could help women feel more in control of their treatment plans and options through shared decision-making processes and understanding [78]. In addition, Reddit users seemed to seek practical information for managing the bleeding as well as information on effective treatments to help with the symptoms. Therefore, better training and resource allocation for health care professionals would allow them to provide accurate and appropriate support in managing this condition [14].

### Strengths and Limitations

While this study provides a unique insight into the experiences of Reddit users with HMB, several limitations must be acknowledged. First, data were drawn exclusively from an English language forum and therefore may limit the contribution of other non–English-speaking groups of users who may experience HMB in a different way through cultural or religious beliefs and practices [79]. Second, previous studies have shown that individuals who seek out information for health conditions on the internet and engage with discussions in online forums tend to be more affluent than those who do not [80]. Therefore, the findings may not adequately reflect the experiences of low-income individuals or those experiencing period poverty, who may face additional barriers.

A third limitation is that our search was restricted to specific terms related to HMB and excluded named conditions such as fibroids or endometriosis. A broader search could have enhanced the dataset. However, given the difficulty in diagnosing HMB, we feel this would not have substantially added to our findings. We also restricted our search to threads posted within the last 10 years to ensure a focus on contemporary health issues, particularly in light of the significant political changes regarding women’s reproductive health and rights during this period.

A fourth and final limitation of this study is the potential for individuals to misrepresent themselves or fabricate experiences online, including falsely claiming to have specific health conditions, which may raise concerns about the authenticity of some posts. However, this was mitigated by our exclusion of posts with <5 unique contributors. It cannot be confirmed that all participants in the study had a formal diagnosis of HMB or were actively experiencing its symptoms. Nonetheless, Reddit users are likely to resemble those individuals who may be in the process of seeking a medical diagnosis or who have a diagnosis [18].

Despite these limitations, this research is the first to examine HMB knowledge sharing and support within online communities. The study captures detailed conversations between 1115 Reddit users on an understudied and heavily stigmatized health condition.

### Future Directions

While this study suggests medical sexism as a barrier to HMB treatment, future research is needed to explore the dynamics of this in relation to HMB, with a focus on the power and discourse around these conversations. While this study discusses this, this was not the focus of this study, and therefore, further exploration is needed. We also recommend that future studies examine how different communities may experience HMB differently through an intersectional lens. In addition, the themes identified in this study should be explored further with offline populations.

### Conclusions

This novel study qualitatively examined the perspectives and experiences of women living with HMB as shared within the Reddit community. The themes demonstrated that Reddit users validated each other’s experiences of HMB and shared practical advice on managing the condition via the sharing of experiential knowledge. However, themes also showed that HMB impacts individuals’ lives beyond a biomedical framework, significantly impacting the quality of life of those experiencing it. Therefore, a biopsychosocial approach to HMB is vital to improving patient outcomes. Finally, Reddit users also discussed medical treatment, including the mixed efficacy of hormone-based treatment, and the inaccessibility of surgical options due to the prioritization of fertility over individual well-being. This study highlights the importance of supportive communities for women experiencing this condition and provides evidence for the potential for online platforms to serve as valuable resources for education, support, and empowerment for women with HMB.

## Appendix

Multimedia Appendix 1 A priori coding template.

## Abbreviations

GP: general practitioner
HBC: hormonal birth control
HMB: heavy menstrual bleeding
IUD: intrauterine device
OP: opening post

## Data Availability

The datasets generated or analyzed during this study are not publicly available due to the sensitive nature of the content and to protect user anonymity but are available from the corresponding author on reasonable request.

## References

[1] Fraser IS, Mansour D, Breymann C, Hoffman C, Mezzacasa A, Petraglia F (2015). Prevalence of heavy menstrual bleeding and experiences of affected women in a European patient survey. Int J Gynaecol Obstet.

[2] Heavy menstrual bleeding: assessment and management. National Institute for Health and Care Excellence.

[3] Friberg B, Kristin Örnö A, Lindgren A, Lethagen S (2006). Bleeding disorders among young women: a population-based prevalence study. Acta Obstet Gynecol Scand.

[4] Heavy periods. National Health Service.

[5] DeLoughery E, Colwill AC, Edelman A, Samuelson Bannow B (2024). Red blood cell capacity of modern menstrual products: considerations for assessing heavy menstrual bleeding. BMJ Sex Reprod Health.

[6] Hapangama DK, Bulmer JN (2016). Pathophysiology of heavy menstrual bleeding. Womens Health (Lond).

[7] Uimari O, Subramaniam KS, Vollenhoven B, Tapmeier TT (2022). Uterine fibroids (Leiomyomata) and heavy menstrual bleeding. Front Reprod Health.

[8] Achanna KS, Nanda J (2022). Evaluation and management of abnormal uterine bleeding. Med J Malaysia.

[9] Huang Q, Liu X, Critchley H, Fu Z, Guo S (2022). How does the extent of fibrosis in adenomyosis lesions contribute to heavy menstrual bleeding?. Reprod Med Biol.

[10] Hussain A, Sehring J, Beltsos A, Shetty MK (2021). Imaging of abnormal uterine bleeding and menstrual disorders. Breast & Gynecological Diseases: Role of Imaging in the Management.

[11] Mansour D, Hofmann A, Gemzell-Danielsson K (2021). A review of clinical guidelines on the management of iron deficiency and iron-deficiency anemia in women with heavy menstrual bleeding. Adv Ther.

[12] Duckitt K (2015). Menorrhagia. BMJ Clin Evid.

[13] Kuzmina N, Palmblad J, Mints M (2011). Predictive factors for the occurrence of idiopathic menorrhagia: evidence for a hereditary trait. Mol Med Rep.

[14] Davies J, Kadir RA (2017). Heavy menstrual bleeding: an update on management. Thromb Res.

[15] Marjoribanks J, Farquhar C, Roberts H, Lethaby A, Lee J (2017). Long-term hormone therapy for perimenopausal and postmenopausal women. Cochrane Database Syst Rev.

[16] Lethaby A, Penninx J, Hickey M, Garry R, Marjoribanks J (2013). Endometrial resection and ablation techniques for heavy menstrual bleeding. Cochrane Database Syst Rev.

[17] Roberts TE, Tsourapas A, Middleton LJ, Champaneria R, Daniels JP, Cooper KG, Bhattacharya S, Barton PM (2011). Hysterectomy, endometrial ablation, and levonorgestrel releasing intrauterine system (Mirena) for treatment of heavy menstrual bleeding: cost effectiveness analysis. BMJ.

[18] da Silva Filho AL, Caetano C, Lahav A, Grandi G, Lamaita RM (2021). The difficult journey to treatment for women suffering from heavy menstrual bleeding: a multi-national survey. Eur J Contracept Reprod Health Care.

[19] Olson MM, Alhelou N, Kavattur PS, Rountree L, Winkler IT (2022). The persistent power of stigma: a critical review of policy initiatives to break the menstrual silence and advance menstrual literacy. PLOS Glob Public Health.

[20] Matteson KA, Raker CA, Clark MA, Frick KD (2013). Abnormal uterine bleeding, health status, and usual source of medical care: analyses using the Medical Expenditures Panel Survey. J Womens Health (Larchmt).

[21] Warner PE, Critchley HO, Lumsden MA, Campbell-Brown M, Douglas A, Murray GD (2004). Menorrhagia I: measured blood loss, clinical features, and outcome in women with heavy periods: a survey with follow-up data. Am J Obstet Gynecol.

[22] van Lonkhuijzen RM, Garcia FK, Wagemakers A (2022). The stigma surrounding menstruation: attitudes and practices regarding menstruation and sexual activity during menstruation. Women Reprod Health.

[23] Johnston-Robledo I, Chrisler JC, Bobel C, Winkler IT, Fahs B, Hasson KA, Kissling EA, Roberts TA (2020). The menstrual mark: menstruation as social stigma. The Palgrave Handbook of Critical Menstruation Studies.

[24] Wiggleton-Little J (2024). "Just" a painful period: a philosophical perspective review of the dismissal of menstrual pain. Womens Health (Lond).

[25] Henry C, Ekeroma A, Filoche S (2020). Barriers to seeking consultation for abnormal uterine bleeding: systematic review of qualitative research. BMC Womens Health.

[26] Protheroe J, Chew-Graham C (2005). The role of primary care in the diagnosis and management of menorrhagia: a qualitative study of women with menorrhagia. Prim Health Care Res Dev.

[27] Dutton B, Kai J (2023). Women's experiences of heavy menstrual bleeding and medical treatment: a qualitative study in primary care. Br J Gen Pract.

[28] Fox KE (2012). Management of heavy menstrual bleeding in general practice. Curr Med Res Opin.

[29] Sriprasert I, Pakrashi T, Kimble T, Archer DF (2017). Heavy menstrual bleeding diagnosis and medical management. Contracept Reprod Med.

[30] Karlsson TS, Marions LB, Edlund MG (2014). Heavy menstrual bleeding significantly affects quality of life. Acta Obstet Gynecol Scand.

[31] Bitzer J, Serrani M, Lahav A (2013). Women's attitudes towards heavy menstrual bleeding, and their impact on quality of life. Open Access J Contracept.

[32] O'Flynn N (2006). Menstrual symptoms: the importance of social factors in women's experiences. Br J Gen Pract.

[33] Coulson NS, Buchanan H (2022). The role of online support groups in helping individuals affected by HIV and AIDS: scoping review of the literature. J Med Internet Res.

[34] Smailhodzic E, Hooijsma W, Boonstra A, Langley DJ (2016). Social media use in healthcare: a systematic review of effects on patients and on their relationship with healthcare professionals. BMC Health Serv Res.

[35] Garg R, Rebić N, De Vera MA (2020). Information needs about cancer treatment, fertility, and pregnancy: qualitative descriptive study of reddit threads. JMIR Cancer.

[36] Calvi C, Sherman KA, Pham D (2024). Loneliness and perceived social support in endometriosis: the roles of body image disturbance and anticipated stigma. Int J Behav Med.

[37] Sommer M, Hirsch JS, Nathanson C, Parker RG (2015). Comfortably, safely, and without shame: defining menstrual hygiene management as a public health issue. Am J Public Health.

[38] Gabbidon K, Chenneville T (2021). Strategies to minimize further stigmatization of communities experiencing stigma: a guide for qualitative researchers. Stigma Health.

[39] Eysenbach G (2011). Infodemiology and infoveillance: tracking online health information and cyberbehavior for public health. Am J Prev Med.

[40] Johnston EJ, Campbell K, Coleman T, Lewis S, Orton S, Cooper S (2019). Safety of electronic cigarette use during breastfeeding: qualitative study using online forum discussions. J Med Internet Res.

[41] Jiang T, Osadchiy V, Mills JN, Eleswarapu SV (2020). Is it all in my head? Self-reported psychogenic erectile dysfunction and depression are common among young men seeking advice on social media. Urology.

[42] Betts D, Dahlen HG, Smith CA (2014). A search for hope and understanding: an analysis of threatened miscarriage internet forums. Midwifery.

[43] Lindgren S, Richardson L (2023). Endometriosis pain and epistemic community: mapping discourses in online discussions among sufferers. Soc Sci Med.

[44] Ethics guidelines for internet-mediated research. The British Psychological Society.

[45] Eysenbach G, Till J E (2001). Ethical issues in qualitative research on internet communities. BMJ.

[46] King N, Symon G, Cassell C (2012). Doing template analysis. Qualitative Organizational Research: Core Methods and Current Challenges.

[47] Özberk H, Bilgiç D, Badem A (2023). Menstrual cycle abnormalities in women: characteristics, perceptions, and health-seeking behaviours. Eur J Contracept Reprod Health Care.

[48] Chen AT (2014). What's in a virtual hug? A transdisciplinary review of methods in online health discussion forum research. Libr Inf Sci Res.

[49] Mullan K, Béal C, Vanderheiden E, Mayer CH (2021). The use of humour to deal with uncomfortable moments in interaction: a cross-cultural approach. The Palgrave Handbook of Humour Research.

[50] Mora-Ripoll R (2010). The therapeutic value of laughter in medicine. Altern Ther Health Med.

[51] Makoae LN, Greeff M, Phetlhu RD, Uys LR, Naidoo JR, Kohi TW, Dlamini PS, Chirwa ML, Holzemer WL (2008). Coping with HIV-related stigma in five African countries. J Assoc Nurses AIDS Care.

[52] Chapple A, Ziebland S (2004). The role of humor for men with testicular cancer. Qual Health Res.

[53] Cardeña I (2003). On humour and pathology: the role of paradox and absurdity for ideological survival. Anthropol Med.

[54] Kocaoz S, Cirpan R, Degirmencioglu AZ (2019). The prevalence and impacts heavy menstrual bleeding on anemia, fatigue and quality of life in women of reproductive age. Pak J Med Sci.

[55] Garside R, Britten N, Stein K (2008). The experience of heavy menstrual bleeding: a systematic review and meta-ethnography of qualitative studies. J Adv Nurs.

[56] Fahs B (2020). There will be blood: women’s positive and negative experiences with menstruation. Womens Reprod Health.

[57] Skouw-Rasmussen N, Pollard D (2019). Everyday issues in women with bleeding disorders. J Haem Pract.

[58] Moreno Gómez A, Guo P, de la Llave Rincón AI, Efstathiou N (2023). Women's experiences of primary dysmenorrhea symptoms: a systematic review of qualitative evidence and meta-aggregation. Women Health.

[59] Sanigorska A, Chaplin S, Holland M, Khair K, Pollard D (2022). The lived experience of women with a bleeding disorder: a systematic review. Res Pract Thromb Haemost.

[60] Ryan S, Ussher JM, Perz J (2020). Women’s experiences of the premenstrual body: negotiating body shame, self-objectification, and menstrual shame. Womens Reprod Health.

[61] Severinsen C, Neely E, Hutson R (2024). Resisting stigma: the role of online communities in young mothers' successful breastfeeding. Int Breastfeed J.

[62] Nobles AL, Dreisbach CN, Keim-Malpass J, Barnes LE (2018). "Is this a STD? Please help!": online information seeking for sexually transmitted diseases on reddit. Proc Int AAAI Conf Weblogs Soc Media.

[63] Stankowski-Drengler TJ, Tucholka JL, Bruce JG, Steffens NM, Schumacher JR, Greenberg CC, Wilke LG, Hanlon B, Steiman J, Neuman HB (2019). A randomized controlled trial evaluating the impact of pre-consultation information on patients' perception of information conveyed and satisfaction with the decision-making process. Ann Surg Oncol.

[64] Bodegård H, Helgesson G, Olsson D, Juth N, Lynøe N (2021). Shared decision-making in patient–doctor consultations – how does it relate to other patient-centred aspects and satisfaction?. Clin Ethics.

[65] Joosten EA, DeFuentes-Merillas L, de Weert GH, Sensky T, van der Staak CP, de Jong CA (2008). Systematic review of the effects of shared decision-making on patient satisfaction, treatment adherence and health status. Psychother Psychosom.

[66] Seo H, Burkett KM, Okocha M, Ha H, Chaif R, Izhar N, Coelho MB, Jona B, Iqbal A (2025). Social media activism and women's health: endometriosis awareness and support. Digit Health.

[67] Valdez D, Mena-Meléndez L, Crawford BL, Arvind A, Jozkowski KN (2023). Online social media reactions to the overturn of Roe v. Wade: public health implications and policy insights. Sex Res Soc Policy.

[68] Dai Z, Higgs C (2023). Social network and semantic analysis of Roe v. Wade’s reversal on Twitter. Soc Sci Comput Rev.

[69] Lethaby A, Wise MR, Weterings MA, Bofill Rodriguez M, Brown J (2019). Combined hormonal contraceptives for heavy menstrual bleeding. Cochrane Database Syst Rev.

[70] Farquhar C, Brown J (2009). Oral contraceptive pill for heavy menstrual bleeding. Cochrane Database Syst Rev.

[71] Aengst J, Layne LL (2013). The Need to Bleed? A Feminist Technology Assessment of Menstrual-Suppressing Birth Control Pills.

[72] Wemrell M, Gunnarsson L (2023). Claims in the clinic: a qualitative group interview study on healthcare communication about unestablished side effects of the copper IUD. PLoS One.

[73] Becker A (2023). Stratified reproduction, hysterectomy, and the social process of opting into infertility. Gend Soc.

[74] Petterson A, Sutton RM (2017). Sexist ideology and endorsement of men’s control over women’s decisions in reproductive health. Psychol Women Q.

[75] Weyand AC, James PD (2021). Sexism in the management of bleeding disorders. Res Pract Thromb Haemost.

[76] Simona D, Rani S, Raj MA (2023). Biopsychosocial problems faced by women during menopause. Educ Soc.

[77] Zendehdel M, Elyasi F (2018). Biopsychosocial etiology of premenstrual syndrome: a narrative review. J Family Med Prim Care.

[78] Kadir RA, Tarawah A, Shridhar N, Kulkarni R (2024). Driving improvement of diagnosis and awareness of heavy menstrual bleeding in women among physicians. Haemophilia.

[79] Tan DA, Haththotuwa R, Fraser IS (2017). Cultural aspects and mythologies surrounding menstruation and abnormal uterine bleeding. Best Pract Res Clin Obstet Gynaecol.

[80] Chagpar AB (2022). Sociodemographic factors affecting telemedicine access: a population-based analysis. Surgery.

